# Robust Navigation in Multipath Environments Using GNSS/UWB/INS Integration with Anchor Position Estimation Toward eVTOL Operations

**DOI:** 10.3390/s25247419

**Published:** 2025-12-05

**Authors:** Atsushi Osaka, Toshiaki Tsujii

**Affiliations:** Department of Aerospace Engineering, Graduate School of Engineering, Osaka Metropolitan University, Nakamozu Campus, Osaka 599-8531, Japan; sh24361i@st.omu.ac.jp

**Keywords:** GNSS, ultra-wide band, inertial navigation system, positioning accuracy, multipath, signal blockage, urban environment, sensor fusion

## Abstract

Emerging technologies such as urban air mobility and autonomous vehicles increasingly rely on Global Navigation Satellite Systems (GNSS) for accurate positioning. However, GNSS alone suffers from severe degradation in complex environments, particularly due to multipath effects caused by reflections from surrounding structures. These effects distort pseudo-range measurements and, in combination with signal attenuation and blockage, lead to significant positioning errors. To address this challenge, this study proposes a loosely integrated navigation framework that combines GNSS, ultra-wideband (UWB), and inertial navigation system (INS) data. UWB enables high-precision ranging, and we further extend its application to estimate the locations of UWB anchors themselves. This approach alleviates a major technical limitation of UWB systems, which typically require anchor positions near buildings to be precisely surveyed beforehand. Field experiments were conducted in multipath-prone outdoor environments using a drone equipped with GNSS, UWB, and INS sensors. The results demonstrate that the proposed GNSS/UWB/INS integration reduces positioning errors by up to approximately 90% compared with GNSS/INS integration. Moreover, in areas surrounded by UWB anchors (UWB-Anchored Area), submeter-level positioning accuracy was achieved. These findings highlight the robustness of the proposed method against multipath interference and its potential to overcome anchor-dependency issues, thereby contributing to safe and reliable navigation solutions for future urban applications such as eVTOL operations.

## 1. Introduction

The use of GNSS is expanding into a wide range of applications, from consumer devices to critical infrastructure, with eVTOL highlighting the urgent need for enhanced accuracy and reliability. Approximately 10–50 cm of precision is required for eVTOL operations at the Vertiport [1], and in some environments, even greater accuracy is demanded [2]. As the utilization of eVTOLs expands in the future, vertiports will be constructed in various locations, including within urban areas surrounded by buildings [3]. However, GNSS is vulnerable to multipath and signal blockage, both of which significantly degrade positioning accuracy, particularly in urban environments. Multipath, caused by signal reflections, together with signal attenuation due to obstructions, necessitates solutions for robust navigation. GNSS accuracy is generally known to be in the order of several meters [4]. In urban environments, it is expected to deteriorate even further. On the other hand, UWB technology exhibits exceptionally strong characteristics in multipath-prone GNSS environments. UWB enables positioning indoors and outdoors simply by installing multiple devices. Its extremely short pulse width allows for high-precision positioning by combining various techniques such as Two-Way Ranging (TWR), Time Difference of Arrival (TDoA), and Angle of Arrival (AoA). Traditionally, UWB positioning has been widely used indoors, being applied for asset tracking and movement analysis in factories, as well as for analyzing human flow patterns in smart buildings [5,6,7,8]. Compared to Wi-Fi and Bluetooth, which also enable indoor positioning, UWB positioning offers the advantages of high accuracy and low latency. While Wi-Fi positioning accuracy ranges from 1 to several meters and Bluetooth from several tens of centimeters to 1 m [9,10,11,12], UWB has demonstrated positioning accuracy at the submeter level [13]. This is because, unlike Wi-Fi and Bluetooth, which measure distance based on signal strength, UWB uses extremely short pulse waves to measure distance based on the time it takes for the signal to arrive, as mentioned earlier. Furthermore, while Wi-Fi and Bluetooth may take several seconds to determine a location, the UWB positioning process can be completed within one millisecond [8,14]. This is due to its method of simply measuring the round-trip time of a pulse. This demonstrates its suitability for tracking fast-moving objects and applications requiring real-time performance. In recent years, outdoor use has also become widespread, and research on outdoor positioning targeting the approach and landing phases of drones has been advancing [15,16,17,18]. However, the experiments were conducted in open-sky conditions, which are challenging environments for the simulation of GNSS. In other words, while the number of satellites is low and positioning is unstable in this environment, the effects of multipath waves have not been taken into account. In such an environment, acquiring anchor positions is extremely easy; however, this diverges from the real-world conditions where Vertiports will be installed in the future. Furthermore, experiments in multipath environments have focused on positioning for ground-based mobility and people. That is, they targeted subjects exhibiting significant two-dimensional motion with minimal vertical movement. Also, in these papers, the anchor positions were determined precisely by RTK, etc.; therefore, the anchor positioning methodology was out of their research scope.

**Figure 1:**
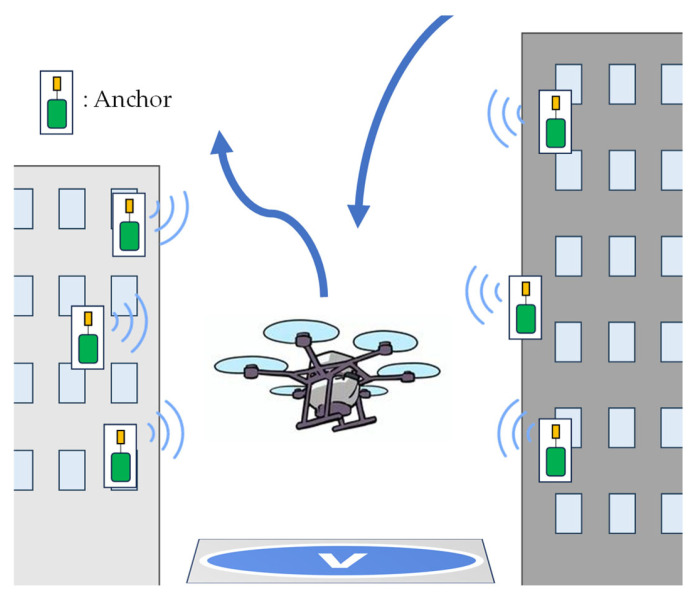
Image of a Vertiport surrounded by UWB anchors.

This study addresses the GNSS challenges of Vertiports by developing robust GNSS/UWB/INS-integrated navigation in multipath environments such as urban areas. Vertiports are expected to be deployed in phases in the following environments: ① open skies, ② urban areas with GNSS multipath conditions, and possibly ③ underground or indoor locations. This experiment focused on the second stage: an outdoor multipath environment in an urban area. In the experiment, we simulated a vertiport surrounded by small buildings in an urban area, as shown in Figure 1, using a drone as the positioning target. Furthermore, in addition to implementing the integrated navigation, we propose a method of anchor position estimation, which we refer to as “the inverted UWB” in this paper. The inverted UWB method eliminates the need for the precise prior positioning of anchors by GNSS-RTK, thereby facilitating the construction of portable vertiports and enhancing the utility of eVTOLs.

This paper is structured as follows: Section 1 outlines the research background and objectives; Section 2 details UWB ranging; Section 3 details the anchor position estimation method; Section 4 presents an overview of GNSS/UWB/INS combined navigation; Section 5 describes outdoor experiments in multipath environment; Section 6 summarizes the findings and discusses future work.

## 2. Range Estimation Using Time of Flight Technology

The following shows the principle of range estimation. The principle of Two-Way Ranging distance measurement used in this study is described below [19]. Other methods of UWB ranging include signal strength and signal reception time differences, but these will not be discussed here.

In the Time-of-Flight method, signals are sent and received between devices, and the distance is calculated by measuring the communication time. The time required from transmission to reception is called the Time of Flight. UWB allows short pulse signals to be used, resulting in high temporal resolution and ranging accuracy.

**Figure 2:**
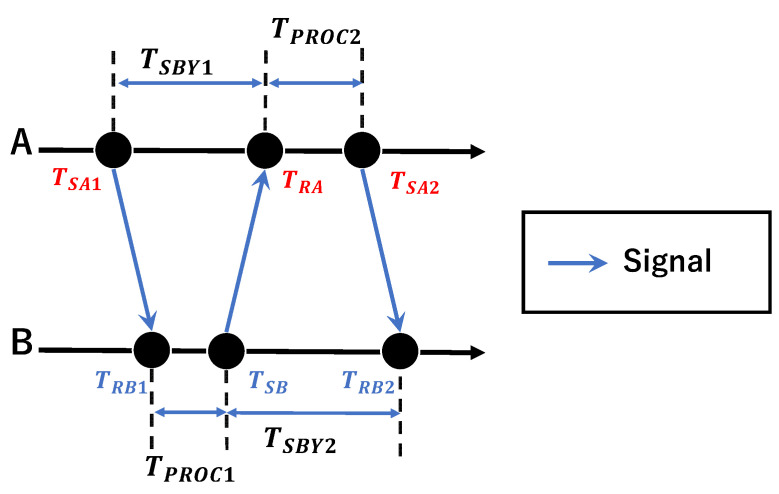
Conceptual diagram of Double-Sided Two-Way Ranging. A indicates a tag, and B indicates an anchor.

A conceptual diagram of Double-Sided Two-Way Ranging (DS-TWR) is shown in Figure 2. In this method, one more transmission/reception is added to the Single-Sided Two-Way Ranging method. The message transmission/reception times for Device A are $T_{SA1}$, $T_{RA}$, and $T_{SA2}$, and the times for Device B are $T_{RB1}$, $T_{SB}$, and $T_{RB2}$. From Figure 2, the signal transmission and reception interval for each device is as follows:

(1) $$T_{SBY1}=T_{RA}-T_{SA1},$$

(2) $$T_{PROC1}=T_{SB}-T_{RB1},$$

(3) $$T_{SBY2}=T_{RB2}-T_{SB}$$

(4) $$T_{PROC2}=T_{SA2}-T_{RA}.$$

There are various methods for calculating the distance d between devices, one of which is expressed using the speed of light c, as shown in Equation (5).

(5) $$d=c\frac{T_{SBY1}\cdot T_{SBY2}-T_{PROC1}\cdot T_{PROC2}}{T_{SBY1}+T_{SBY2}+T_{PROC1}+T_{PROC2}}.$$

Assuming that $e_{a}$ and $e_{b}$ are error terms induced by clock drift, each period is determined as in Equation (6).

(6) $$\left\{\begin{array}{c} {\hat{T}}_{SBY1}=\left(1+e_{a}\right)T_{SBY1}=k_{a}T_{SBY1}\\ {\hat{T}}_{PROC1}=\left(1+e_{b}\right)T_{PROC1}=k_{b}T_{PROC1}\\ {\hat{T}}_{SBY2}=\left(1+e_{b}\right)T_{SBY2}=k_{b}T_{SBY2}\\ {\hat{T}}_{PROC2}=\left(1+e_{a}\right)T_{PROC2}=k_{a}T_{PROC2} \end{array}\right.$$

The communication time (${\hat{T}}_{flight}$) between devices, including clock drift, is calculated as follows. Assuming that the processing time is the same for devices A and B, and that the distance between devices does not change during the series of signal exchanges, we obtain Equations (7) and (8).

$${\hat{T}}_{flight}=\frac{{\hat{T}}_{SBY1}\cdot {\hat{T}}_{SBY2}-{\hat{T}}_{PROC1}\cdot {\hat{T}}_{PROC2}}{{\hat{T}}_{SBY1}+{\hat{T}}_{SBY2}+{\hat{T}}_{PROC1}+{\hat{T}}_{PROC2}}$$

(7) $$\;\;\;\;\;\;=\frac{{\hat{T}}_{SBY1}\cdot {\hat{T}}_{SBY2}-{\hat{T}}_{PROC1}\cdot {\hat{T}}_{PROC2}}{2\left({\hat{T}}_{SBY1}+{\hat{T}}_{PROC2}\right)}$$

(8) $$\;\;\;\;\;\;=\frac{{\hat{T}}_{SBY1}\cdot {\hat{T}}_{SBY2}-{\hat{T}}_{PROC1}\cdot {\hat{T}}_{PROC2}}{2\left({\hat{T}}_{SBY2}+{\hat{T}}_{PROC1}\right)}$$

Substituting Equation (6) into Equations (7) and (8), respectively, we obtain Equations (9) and (10).

(9) $${\hat{T}}_{flight}=\frac{k_{a}k_{b}}{k_{a}}\frac{T_{SBY1}\cdot T_{SBY2}-T_{PROC1}\cdot T_{PROC2}}{2\left(T_{SBY1}+T_{PROC2}\right)}=k_{b}T_{flight}$$

(10) $$=\frac{k_{a}k_{b}}{k_{b}}\frac{T_{SBY1}\cdot T_{SBY2}-T_{PROC1}\cdot T_{PROC2}}{2\left(T_{SBY2}+T_{PROC1}\right)}=k_{a}T_{flight}.$$

We now consider the effects of incorporating these clock drifts. Equations (11) and (12) illustrate the errors introduced by including clock drift. Here, $e_{a}$ and $e_{b}$ are extremely small, at most $\pm 20\;\left[ppm\right]$, and can be largely ignored. In other words, this range estimation method does not require consideration of clock drift.

(11) $${\hat{T}}_{flight}-T_{flight}=k_{a}T_{flight}-T_{flight}=e_{a}T_{flight}$$

(12) $${\hat{T}}_{flight}-T_{flight}=k_{b}T_{flight}-T_{flight}=e_{b}T_{flight}$$

## 3. Anchor Position Estimation

In UWB positioning, the tag’s position is often determined within an area enclosed by anchors. Anchor positions must be known before UWB positioning. Furthermore, the accuracy of anchor positions significantly impacts the positioning accuracy of moving objects. However, when used outdoors, anchors are often installed near buildings, making it difficult to directly obtain anchor positions via GNSS. Therefore, in this research, we propose a method called the inverted UWB method for estimating anchor positions with known tag positions. This is analogous to “inverted pseudolite” [20,21]. While the usual pseudolite positioning method estimates the receiver’s position, the inverted pseudolite assumes the receiver’s position is known and estimates the pseudolite’s (transmitter’s) position. Similarly, the inverted UWB assumes the trajectory of the tag is known, and positions of anchors are estimated. In this study, the positions of the tag are estimated by GNSS-RTK and are treated as the true trajectory. The following describes the algorithm of the inverted UWB.

In this chapter, position coordinates are expressed in the ENU coordinate system. The position $p_{k}$ of the tag obtained from GNSS at time $t_{k}$ is defined by Equation (13). The position of the i-th anchor $a_{i}$ is defined by Equation (14) and this is an unknown quantity. We estimated the position of each anchor separately one by one. Here, $M_{i}$ is the number of observations obtained for i-th anchor and N is the number of anchors. Then, the range between the anchor and tag is calculated from Equation (15).

(13) $$p_{k}={\left[\begin{array}{ccc} X_{k} & Y_{k} & Z_{k} \end{array}\right]}^{T},\;\;\left(k=1,\ldots \;,\;M_{i}\right),$$

(14) $$a_{i}={\left[\begin{array}{ccc} x_{i} & y_{i} & z_{i} \end{array}\right]}^{T},\;\;(i=1,\;\ldots \;,\;N),$$

(15) $$h_{i,\;k}\left(a_{i}\right)=\left\|p_{k}-a_{i}\right\|=\sqrt{{\left(X_{k}-x_{i}\right)}^{2}+{\left(Y_{k}-y_{i}\right)}^{2}+{\left(Z_{k}-z_{i}\right)}^{2}}$$

The actual observed range $d_{i,\;k}$ contains noise, resulting in the expression given by (16).

(16) $$d_{i,\;k}=h_{i,\;k}\left(a_{i}\right)+v_{i,\;k},$$

(17) $$v_{i,\;k}\sim N\left(0,\;\sigma^{2}\right),$$

For each anchor, the residual vector $r_{i}\left(a_{i}\right)$ between the observed and the computed range is given by Equation (18).

(18) $$r_{i}\left(a_{i}\right)=\left[\begin{array}{c} r_{i,\;1}\\ r_{i,\;2}\\ \vdots \\ r_{i,\;M_{i}} \end{array}\right]=\left[\begin{array}{c} d_{i,\;1}-h_{i,\;1}\left(a_{i}\right)\\ d_{i,\;2}-h_{i,\;2}\left(a_{i}\right)\\ \vdots \\ d_{i,\;M_{i}}-h_{i,\;M_{i}}\left(a_{i}\right) \end{array}\right]$$

The anchor position can be estimated by minimizing the evaluation function $S_{i}$.

(19) $$\underset{a_{i}}{\min}S_{i}\left(a_{i}\right)=\sum_{k=1}^{M_{i}}{\left(r_{i,\;k}\left(a_{i}\right)\right)}^{2}.$$

The Jacobian matrix J of $r_{i,\;k}$ satisfies Equation (20).

$$J=\left[\begin{array}{ccc} \frac{\partial r_{i,\;1}}{\partial x_{i}} & \frac{\partial r_{i,\;1}}{\partial y_{i}} & \frac{\partial r_{i,\;1}}{\partial z_{i}}\\ \frac{\partial r_{i,\;2}}{\partial x_{i}} & \frac{\partial r_{i,\;2}}{\partial y_{i}} & \frac{\partial r_{i,\;2}}{\partial z_{i}}\\ \vdots & \vdots & \vdots \\ \frac{\partial r_{i,\;M_{i}}}{\partial x_{i}} & \frac{\partial r_{i,\;M_{i}}}{\partial y_{i}} & \frac{\partial r_{i,\;M_{i}}}{\partial z_{i}} \end{array}\right]\;\;\;\;\;\;\;\;\;\;\;\;\;\;\;\;\;\;\;\;\;\;\;\;\;\;\;\;\;\;\;\;\;\;\;\;\;\;\;\;\;\;\;\;\;\;\;\;\;\;$$

(20) $$=\left[\begin{array}{ccc} -\frac{X_{1}-x_{i}}{\left\|p_{1}-a_{i}\right\|} & -\frac{Y_{1}-y_{i}}{\left\|p_{1}-a_{i}\right\|} & -\frac{Z_{1}-z_{i}}{\left\|p_{1}-a_{i}\right\|}\\ -\frac{X_{2}-x_{i}}{\left\|p_{2}-a_{i}\right\|} & -\frac{Y_{2}-y_{i}}{\left\|p_{2}-a_{i}\right\|} & -\frac{Z_{2}-z_{i}}{\left\|p_{2}-a_{i}\right\|}\\ \vdots & \vdots & \vdots \\ -\frac{X_{M_{i}}-x_{i}}{\left\|p_{M_{i}}-a_{i}\right\|} & -\frac{Y_{M_{i}}-y_{i}}{\left\|p_{M_{i}}-a_{i}\right\|} & -\frac{Z_{M_{i}}-z_{i}}{\left\|p_{M_{i}}-a_{i}\right\|} \end{array}\right]$$

The Levenberg–Marquardt method was used for anchor position estimation.

(21) $$\Delta a_{i}={\left(J^{T}J+\lambda I\right)}^{-1}J^{T}r_{i}$$

(22) $$a_{i}\leftarrow a_{i}+\Delta a_{i}$$

The initial value of $a_{i}$ was set to the average value of $p_{k}$, that is, the average coordinates of the flight path. λ is the coefficient that controls convergence.

## 4. GNSS/UWB/INS Combined Navigation Overview

**Figure 3:**
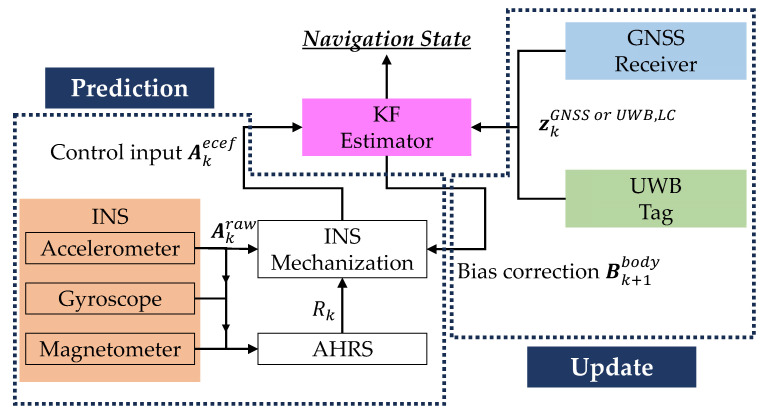
System configuration diagram of the loosely coupled method.

**Figure 4:**
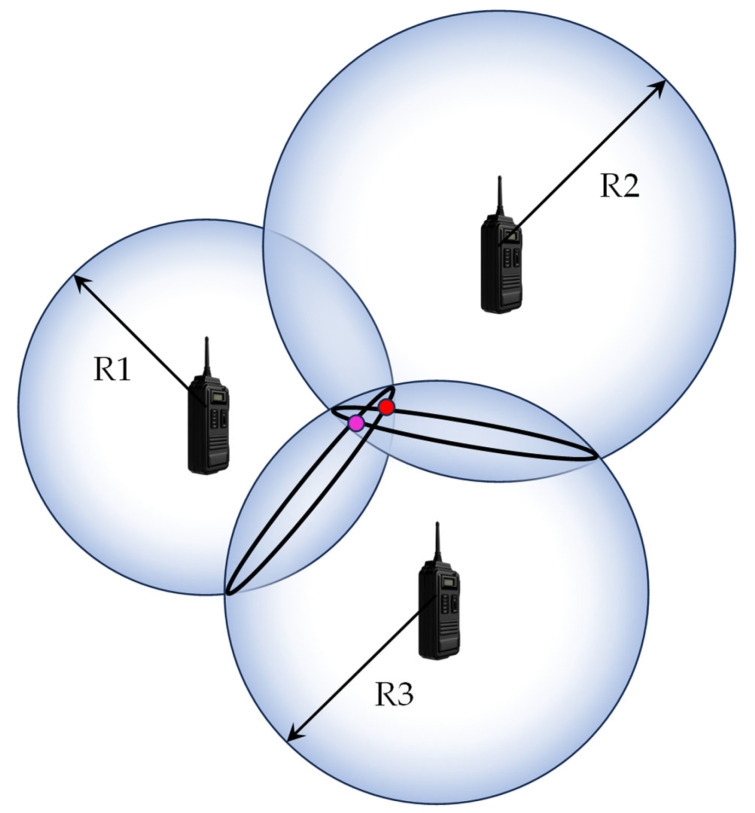
UWB position estimation based on 3D trilateration.

Standalone INS struggles with accumulated errors, especially with low-cost INSs, and in GNSS-denied areas, accuracy degrades even when using GNSS/INS. UWB offers a promising solution, providing precise position relative to anchors even in environments that are challenging for GNSS. By integrating GNSS and UWB with INS, the strengths of each system are combined, significantly improving overall navigation accuracy. This section describes the loosely coupled integration method we applied [22]. Figure 3 shows the system configuration for the loosely coupled integration method. The state equation in the Kalman filter is the simple INS equation, and the observation equation is composed of the position output from the GNSS and UWB. GNSS positioning involves four unknowns—three position coordinates and a clock error—so it requires signals from at least four GNSS satellites. Similarly, as shown in Figure 4, three anchors cannot isolate the solution to a single point for UWB positioning [23]. To obtain a unique positioning solution, communication with at least four anchors is required. While the GNSS/UWB ranging data are treated as the measurements in the tightly coupled integration, the position solutions by GNSS/UWB are treated as the measurements in loosely coupled integration, making the algorithm simpler.

- State Equation

Three-dimensional position coordinates of the UWB Tag in the fixed earth coordinate system $X_{k}^{ecef}={\left[\begin{array}{ccc} x_{k}^{ecef} & y_{k}^{ecef} & z_{k}^{ecef} \end{array}\;\right]}^{T}$, velocity $V_{k}^{ecef}={\left[\begin{array}{ccc} v_{x,k}^{ecef} & v_{y,k}^{ecef} & v_{z,k}^{ecef} \end{array}\right]}^{T}$ and the acceleration bias in the aircraft coordinate system $B_{k}^{body}={\left[\begin{array}{ccc} b_{x,k}^{body} & b_{y,k}^{body} & b_{z,k}^{body} \end{array}\right]}^{T}$ define the state vector as in Equation (23).

(23) $$x_{k}^{LC}={\left[\begin{array}{ccc} X_{k}^{ecef} & V_{k}^{ecef} & B_{k}^{body} \end{array}\right]}^{T}$$

The acceleration obtained from the INS is in the aircraft body coordinate system as in Equation (24). In contrast, the position and velocity in the state $x_{k}^{LC}$ are in the earth-fixed coordinate system, and therefore the acceleration must be transformed as in Equation (25). $R_{LB}$ is the transformation matrix from the airframe coordinate system to the local coordinate system, and $R_{GL}$ is the transformation matrix from the local coordinate system to the earth-fixed coordinate system. $R_{k}$ in Figure 3 corresponds to $R_{GL}\;R_{LB}$.

(24) $$A_{k}^{raw}={\left[\begin{array}{ccc} a_{x,\;k}^{body} & a_{y,\;k}^{body} & a_{z,\;k}^{body} \end{array}\right]}^{T}$$

(25) $$A_{k}^{ecef}=R_{GL}R_{LB}\left(A_{k}^{raw}-B_{k}^{body}\right)$$

Using $x_{k}^{LC}$, the equation of state can be expressed as in Equation (26). $w_{k}$ denotes the Gaussian noise vector.

(26) $$x_{k}^{LC}=f\left(x_{k-1}^{LC},\;A_{k}^{ecef}\right)+w_{k}$$

The state transition function f can be expressed as in Equation (27). The constant velocity model is used for the position, and the acceleration obtained from the INS is used to calculate the velocity.

(27) $$f\left(x_{k-1}^{LC},\;A_{k}^{ecef}\right)=\left[\begin{array}{c} x_{k-1}^{ecef}+v_{x,\;k-1}^{ecef}\cdot \Delta t\\ y_{k-1}^{ecef}+v_{y,\;k-1}^{ecef}\cdot \Delta t\\ z_{k-1}^{ecef}+v_{z,\;k-1}^{ecef}\cdot \Delta t\\ v_{x,\;k-1}^{ecef}+a_{x,\;k-1}^{ecef}\cdot \Delta t\\ v_{y,\;k-1}^{ecef}+a_{y,\;k-1}^{ecef}\cdot \Delta t\\ v_{z,\;k-1}^{ecef}+a_{z,\;k-1}^{ecef}\cdot \Delta t\\ b_{x,\;k-1}^{body}\\ b_{y,\;k-1}^{body}\\ b_{z,\;k-1}^{body} \end{array}\right]$$

- Observation Equation

The positioning solution in the ECEF coordinate system obtained from GNSS and/or UWB is the measurement (Equation (28)) for the positioning filter integrated with INS. Using this, the observation equation is expressed as in Equation (29).

(28) $$z_{k}^{LC}=\left[\begin{array}{ccc} x_{k}^{GNSS\;or\;UWB} & y_{k}^{GNSS\;or\;UWB} & z_{k}^{GNSS\;or\;UWB} \end{array}\right]$$

(29) $$z_{k}^{LC}=h^{LC}\left(x_{k}^{LC}\right)+v_{k}^{LC}$$

The observation function $h^{LC}$ satisfies Equation (30) and $v_{k}^{LC}$ is Gaussian noise vector.

(30) $$h^{LC}\left(x_{k}^{LC}\right)=X_{k}^{ecef}=\left[\begin{array}{c} x_{k}^{ecef}\\ y_{k}^{ecef}\\ z_{k}^{ecef} \end{array}\right]$$

When updates are performed using GNSS data, the observation noise covariance matrix $R_{k}$ is defined as dependent on both Dilution of Precision (DOP) and the User Equivalent Ranging Error (UERE). On the other hand, when updates are performed using UWB data, it is defined as dependent solely on UERE. This is because the positioning solution obtained via UWB is considered significantly more accurate and less noisy than the GNSS positioning solution. The UERE for GNSS observations is denoted as $s_{UERE}^{GNSS}$, and the UERE for UWB observations is denoted as $s_{UERE}^{UWB}$.

## 5. Outdoor Positioning Experiment

The following is a description of the outdoor positioning experiments conducted in this study.

### 5.1. Experimental Objective and Methods

**Figure 5:**
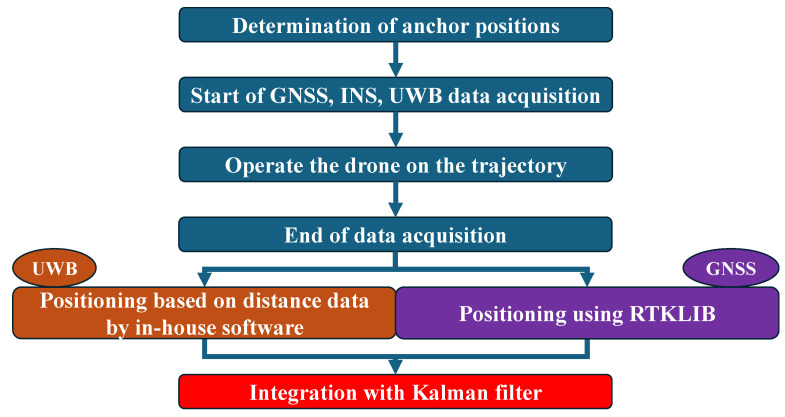
Experimental flow.

In this study, outdoor positioning experiments using a drone were conducted to implement GNSS/UWB/INS integrated navigation in a multipath environment. Figure 5 shows the flowchart of the experiment. After positioning the anchor, data acquisition is initiated by various devices mounted on the drone. The drone then flies along the planned route. For UWB, positioning is performed by Extended Kalman Filter (EKF) method [24] using our in-house software EKF-omugnss ver 1.0, and for GNSS, positioning is performed using RTKLIB ver 2.4.2 [25], and a loosely coupled integrated calculation is performed using the positioning solutions and INS data.

### 5.2. Experimental Setup

**Figure 6:**
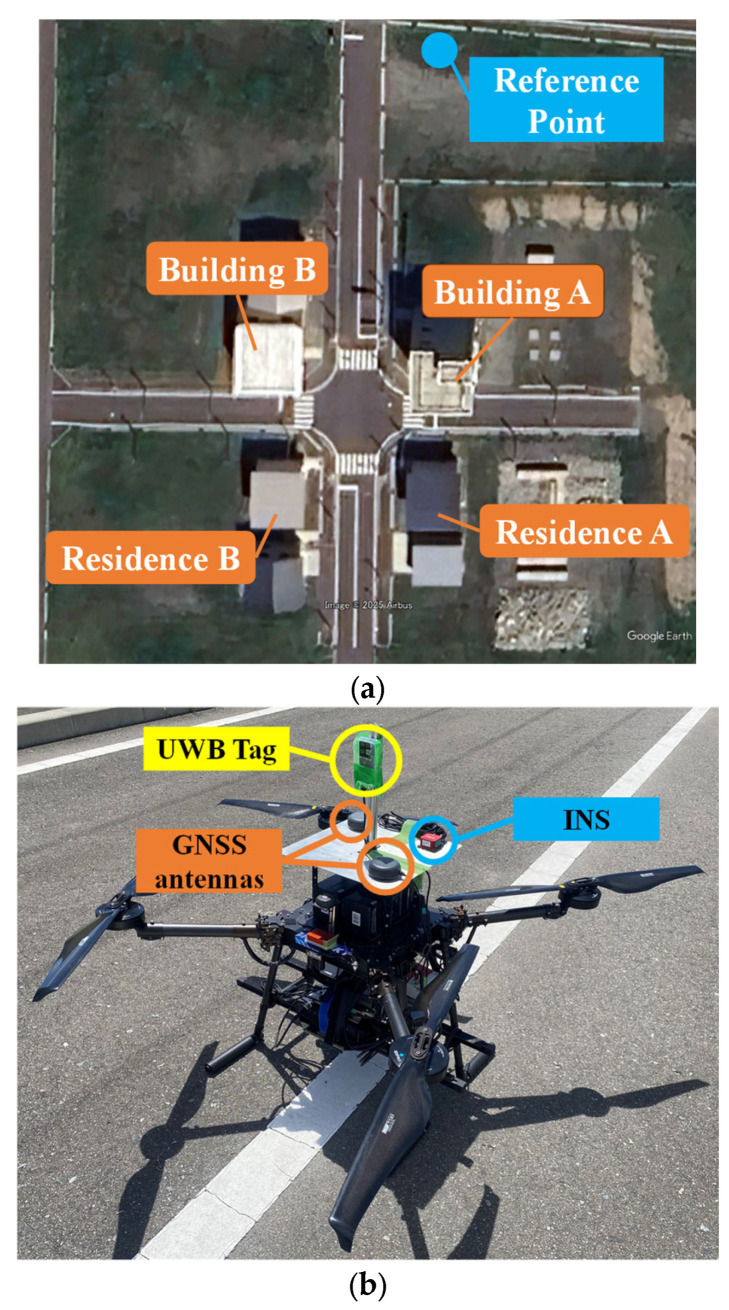
(**a**) Appearance of the experiment site. (Image: Google Earth Pro, © 2025 Google.) This is a satellite photo of the Fukushima RTF urban field. (**b**) The drone used in the experiment. Two GNSS antennas, INS and UWB devices, are mounted on the top of the drone.

Figure 6 shows a satellite image of the Fukushima Robot Test Field (Fukushima RTF) urban field where the experiment was conducted. Building A is a five-story building, and Building B, Residence A and B are two-story buildings. Therefore, the intersection surrounded by these buildings is a multipath-rich environment with poor GNSS reception. The blue dot indicates the position of the reference station used for GNSS relative positioning, and the coordinates are provided by Fukushima RTF ($lat:\;37{}^{\circ}\;37^{\prime }\;53.522^{\prime \prime }$, $lon:\;141{}^{\circ}\;00^{\prime }\;53.778^{\prime \prime }$, $alt:\;4.7\;\left[m\right]$) [26].

Figure 6 also shows the appearance of the drone. The drone is an FP29-10, which was owned and operated by Tokyo Aircraft Instrument Co., Ltd. (Tokyo, Japan). The drone is equipped with a UWB device (Murata Manufacturing, Kyoto, Japan: Type 2BP EVK), two triple-frequency patch antennas (Calian, Ottawa, ON, Canada: TW7972), GNSS receivers (NovAtel, Calgary, AB, Canada: PwrPak7, Septentrio, Leuven, Belgium: AsteRx-i3 D pro+, mosaic-go, ublox, Thalwil, Switzerland: EVK-M8T), INSs (SBG, Carrieres-sur-Seine, France: Ellipse-E, Septentrio: AsteRx-i3 D pro+). The most high-end GNSS receiver, PwrPak7, was used to generate the drone’s reference trajectory. The data of EVK-M8T (the most low-end GNSS receiver) is used for integrated navigation.

**Figure 7:**
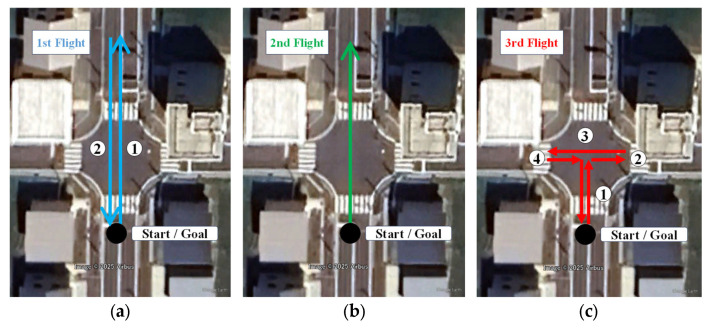
Flight route. (**a**) First flight; (**b**) second flight; (**c**) third flight. (Image: Google Earth Pro, ©2025 Google).

Figure 7 shows the flight paths. The first flight followed a route heading north then south, the second flight only headed north, and the third flight followed the route shown on the right side of Figure 7.

**Figure 8:**
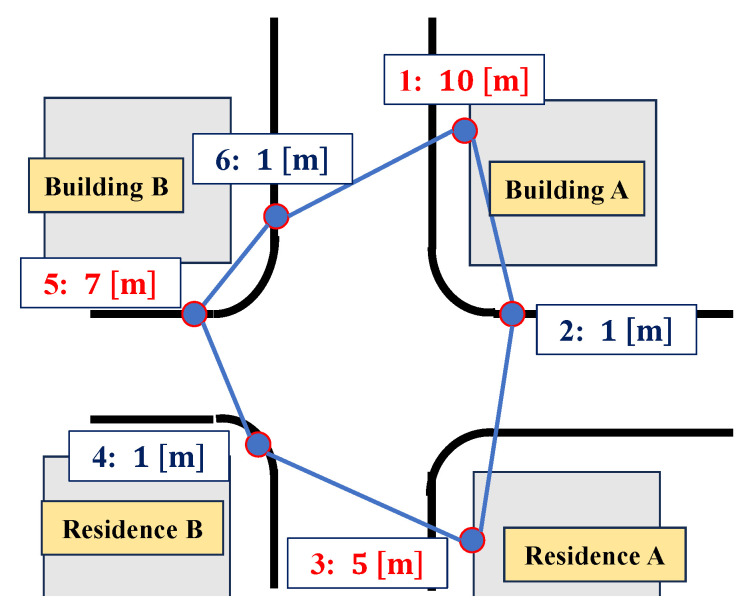
Anchor placement (anchor no.: height).

**Figure 9:**
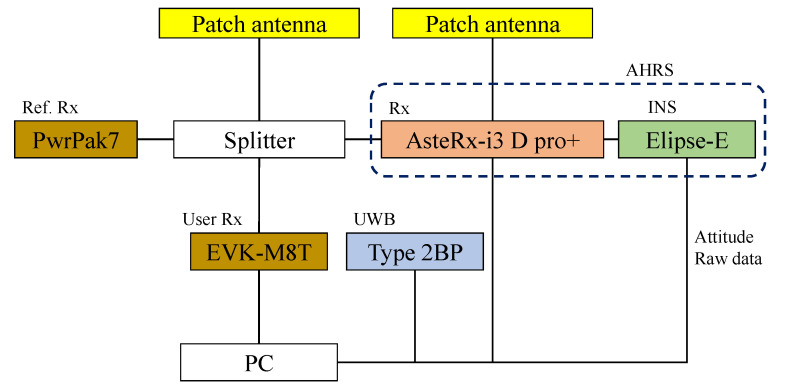
Connection diagram of equipment mounted on the drone.

Figure 8 shows the placement of the anchors. The anchors were numbered from 1 to 6 for identification and installed near buildings. As shown in the figure, anchors 2, 4, and 6 were positioned at similar heights, while anchors 1, 3, and 5 were positioned at different heights to improve the DOP in the upward direction. The connection diagrams of the devices on the drone are shown in Figure 9. Two GNSS antennas were used for attitude angle estimation in the AHRS. The parameters of UERE were defined as shown in Table 1. The UERE for GNSS observations was set to 15 if the PDOP was greater than five, 10 if greater than three, and 8 if three or less. For UWB observations, however, the UERE was fixed at 0.1 regardless of the UWB DOP.

### 5.3. Results and Analysis

Table 2 shows the anchor positions used in the calculations. We conducted three flight tests, and these anchor coordinates were estimated from the data of the third flight.

**Figure 10:**
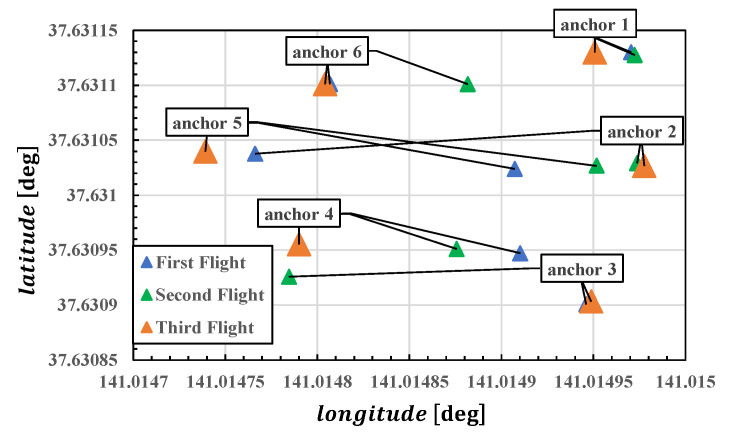
Horizontal anchor positions obtained from data for each flight.

Here, we consider the impact of flight paths on anchor position estimation. We were able to calculate the anchor position using the data from the first and second flights. However, as shown in Figure 10, the estimated results from the first and second flights deviated significantly from the true value due to the influence of the flight path. Table 3 shows the anchor position estimation error in the ENU direction. Here, the error comparison reference is the anchor position estimated from data for the third flight. As shown in Figure 7, the first and second movements were almost exclusively in the north–south direction. Consequently, the east–west constraints weakened, likely resulting in larger estimation errors compared to the north–south direction. Additionally, the vertical direction, even during the flight, did not involve movements as large as those in the horizontal direction, resulting in slightly unstable estimation results.

**Figure 11:**
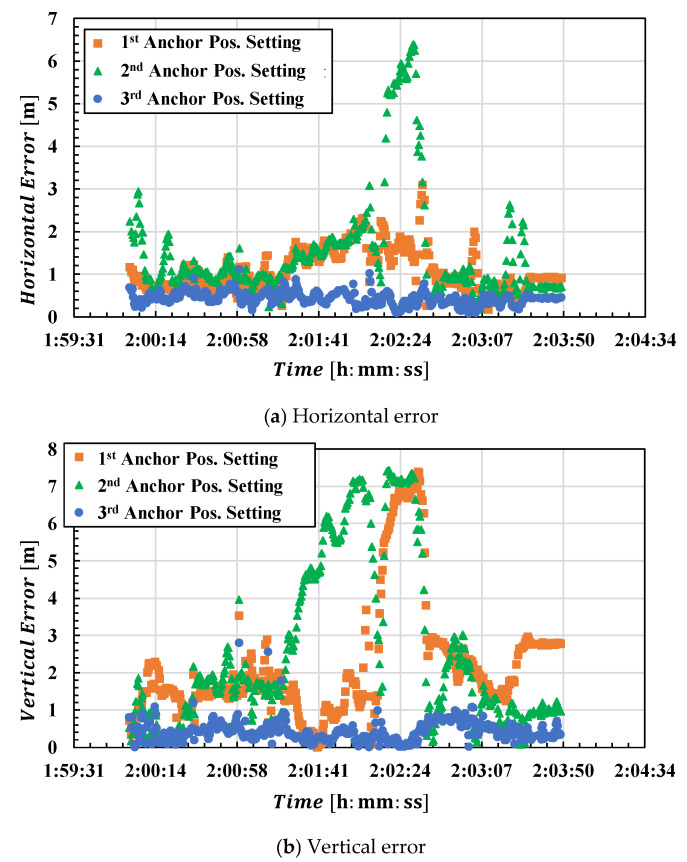
Plot of UWB positioning errors for the third flight. (**a**): horizontal error; (**b**): vertical error. The orange line shows the error calculated using anchor positions estimated from data for the first flight (1st Anchor Position Setting), the green line shows the error for the 2nd Anchor Position Setting, and the blue line shows the error for the 3rd Anchor Position Setting.

Using the three anchor position estimates shown in Figure 10, we performed UWB standalone positioning for the drone during the third flight. Figure 11 shows the horizontal (a) and vertical (b) errors for each anchor position setting. The reference values used for error calculation were obtained from the relative positioning results using a high-end receiver and a ground-reference receiver. Positioning results using anchor positions calculated from data for the first and second flights exhibited significantly large errors. The time when positioning errors began to increase was close to when the drone started moving east–west. This can be considered consistent with the fact that the anchor positions were calculated from data for the first and second flights, which only involved north–south movements, resulting in weaker east–west constraints. The errors were particularly large when using the second anchor estimation result, which followed a northbound-only path.

Furthermore, in the vertical direction, the error has a significant impact because the height difference between anchors was not large. That is, the same anchor position error has a greater effect on the vertical direction than on the horizontal direction. The figure clearly shows that disruption of the vertical geometric relationship ultimately leads to larger mobile positioning errors.

Based on the above results, subsequent positioning calculations will use data obtained from the third flight.

**Figure 12:**
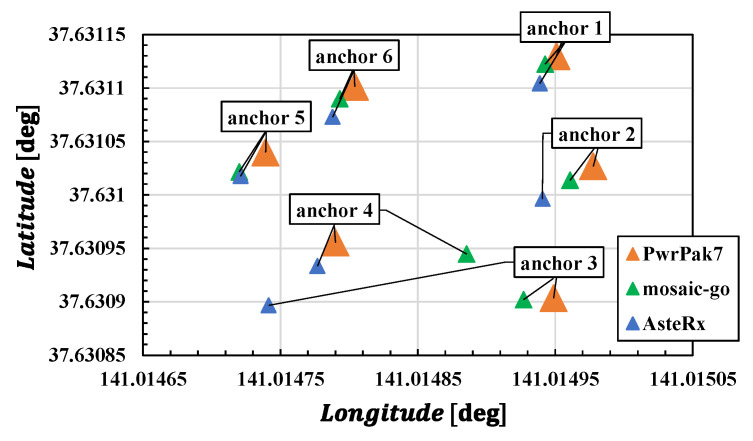
Horizontal anchor positions obtained by using each receiver data.

Next, we will consider the impact of tag trajectory accuracy on anchor position estimation. To this end, we will consider anchor positions estimated using the low-accuracy trajectories of the tag obtained from lower-cost receivers’ data. This situation would simulate a challenging multipath environment because multipath mitigation performance depends on the quality of the receiver. Figure 12 shows the estimated anchor positions using tag trajectories obtained from the high-end receiver (PwrPak7) and those obtained from the low-cost receivers (AsteRx-i3 D pro+, mosaic-go). Table 4 shows the tag position estimation error (RMSE) and the number of solutions obtained using low-cost receivers compared to those from PwrPak7. Both solutions had errors of several meters relative to PwrPak7 data. For mosaic-go data, the RMSE was smaller than that for AsteRx-i3 D pro+ data, but the number of solutions was approximately half. Table 5 shows the error in the anchor position estimated from low-cost receiver data in the ENU coordinate system compared to the anchor position estimated from PwrPak7 data.

**Figure 13:**
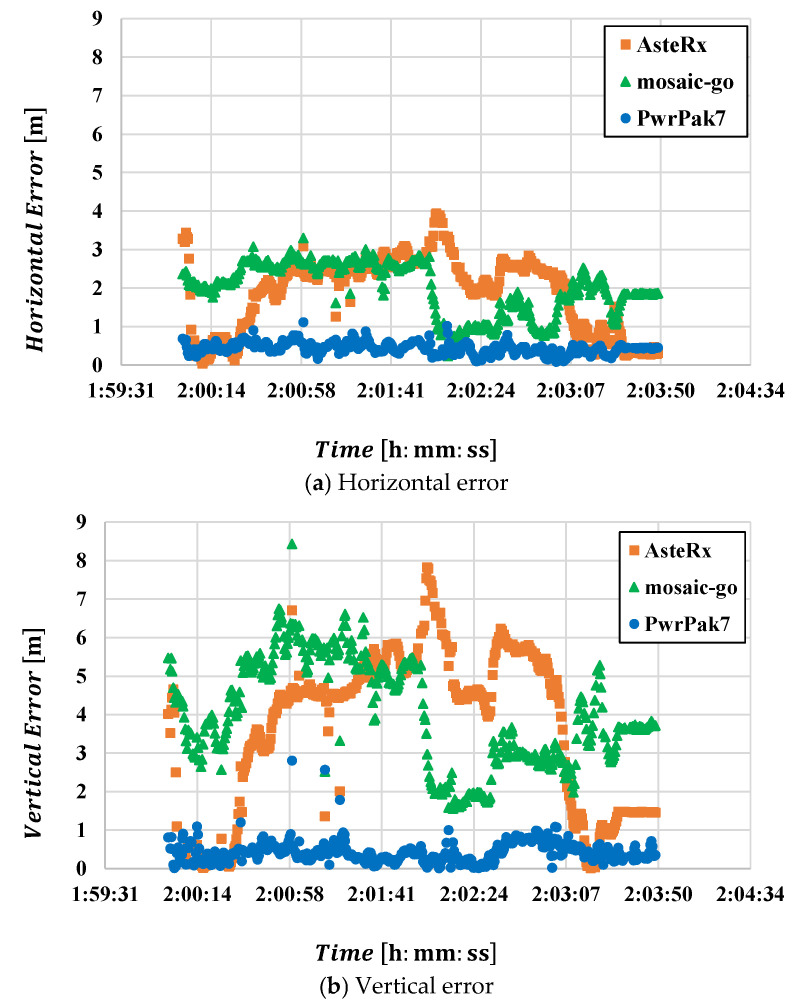
Plot of UWB positioning errors for the third flight. (**a**): horizontal error; (**b**): vertical error. The orange line shows the error calculated using anchor positions estimated from the AsteRx-i3 D pro+ data, the green line shows the error calculated using anchor positions estimated from the mosaic-go data, and the blue line shows the error calculated using anchor positions estimated from the PwrPak7 data.

Similarly to Figure 11, Figure 13 shows the error when performing UWB standalone positioning using the estimated anchor positions derived from the data received from each receiver. The figure shows that the accuracy of the GNSS receiver used for anchor position estimation and the reception environment significantly impact UWB standalone positioning. Notably, the vertical positioning error was significant. This is thought to be influenced by the large vertical positioning error inherent to GNSS. As shown in Table 5, the vertical error was the largest. Consequently, the estimated anchor positions also exhibited substantial vertical deviations, significantly affecting the UWB positioning solution.

**Figure 14:**
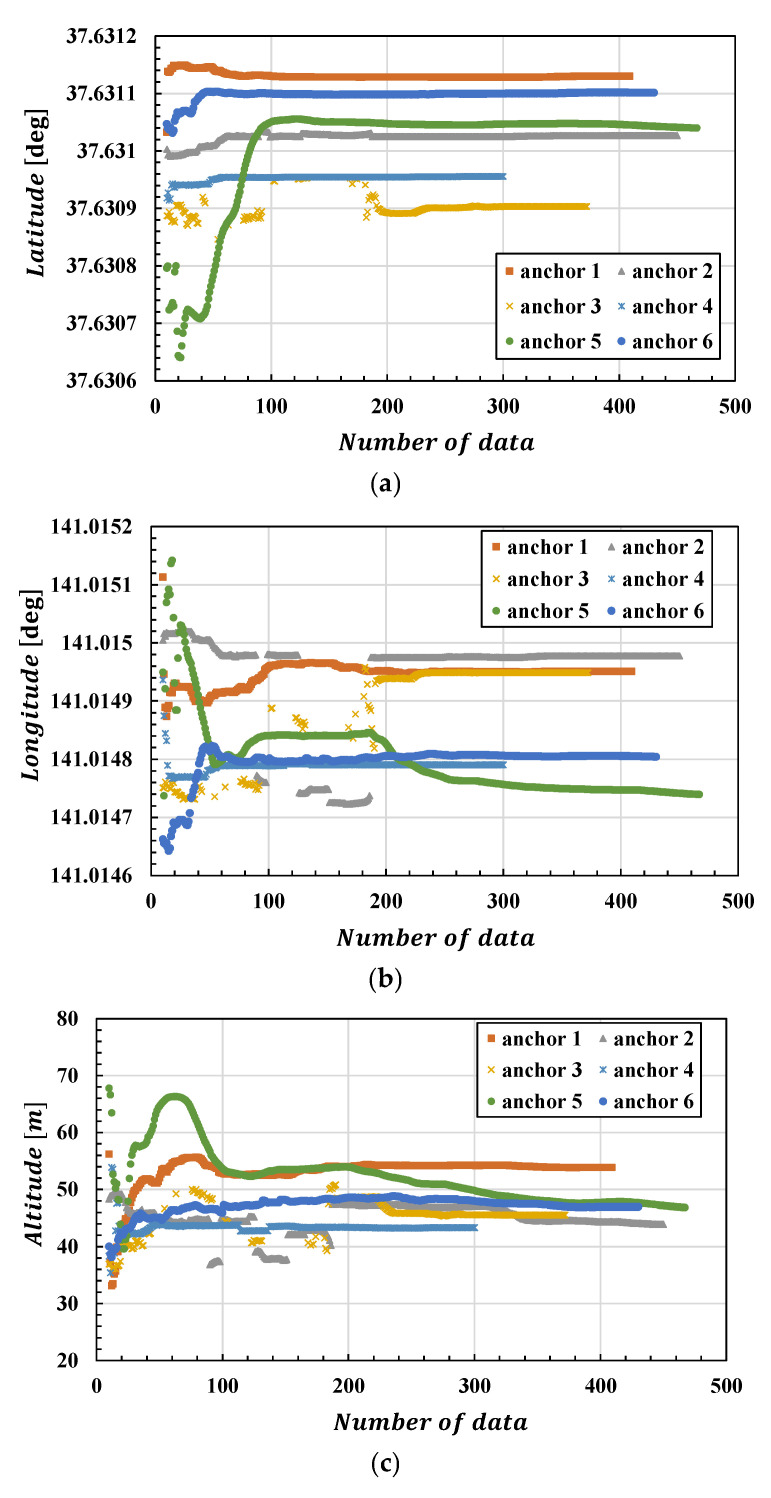
Change in estimated anchor position ((**a**) latitude; (**b**) longitude; (**c**) altitude) in relation to the number of data used for the estimation.

Also, we considered the convergence of anchor position estimation by the inverted UWB. Figure 14 shows how estimation results change with the number of data used for the estimation. This figure shows latitude, longitude, and altitude from top to bottom. Generally, after the number of data exceeds 200, estimation results show little fluctuation and can be considered converged. However, this depends on the quality of the acquired range data, and the occurrence of significant temporal fluctuations in the estimation results, as seen with Anchor 2, still remains. To avoid this, it is desirable that, during flight, when estimating the anchor position, the environment between the UWB antennas is free of obstacles and allows for uninterrupted ranging. Furthermore, convergence time is also thought to depend on the target flight path. By moving significantly up, down, left, and right in the UWB-Anchored Area, it becomes possible to estimate the anchor position from multiple directions, enabling more accurate estimation. In this experiment, there was an anchor whose initial value (the GNSS-RTK mean value) was significantly distant from the convergence value. Had a closer position been used as the initial value, convergence would likely have been faster, enabling the estimation of a reliable anchor position using a shorter data period.

**Figure 15:**
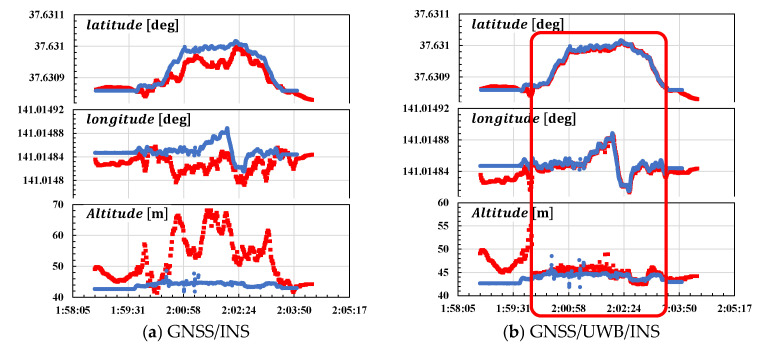
Experimental results in the ENU coordinate system. Red indicates integration results and blue indicates reference data. (**a**) Results of GNSS/INS; (**b**) results of GNSS/UWB/INS.

Figure 15 shows the third positioning result. In the integrated navigation system, GNSS data is obtained from EVK-M8T, the lowest-cost receiver, as previously described, while INS data is obtained from Ellipse-E. The GNSS/INS-integrated results (left) and GNSS/UWB/INS-integrated results (right) are shown as red lines, while the reference values are shown as blue lines. UWB positioning was performed during the period enclosed in red boxes. For the horizontal and vertical directions, the UWB integration significantly reduces errors in multipath environments.

**Figure 16:**
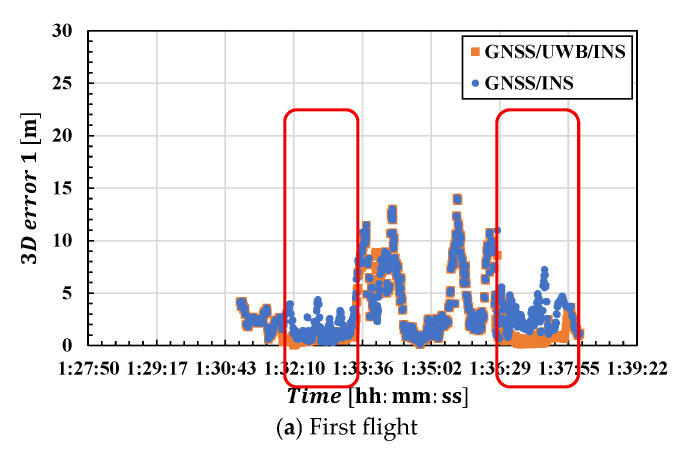

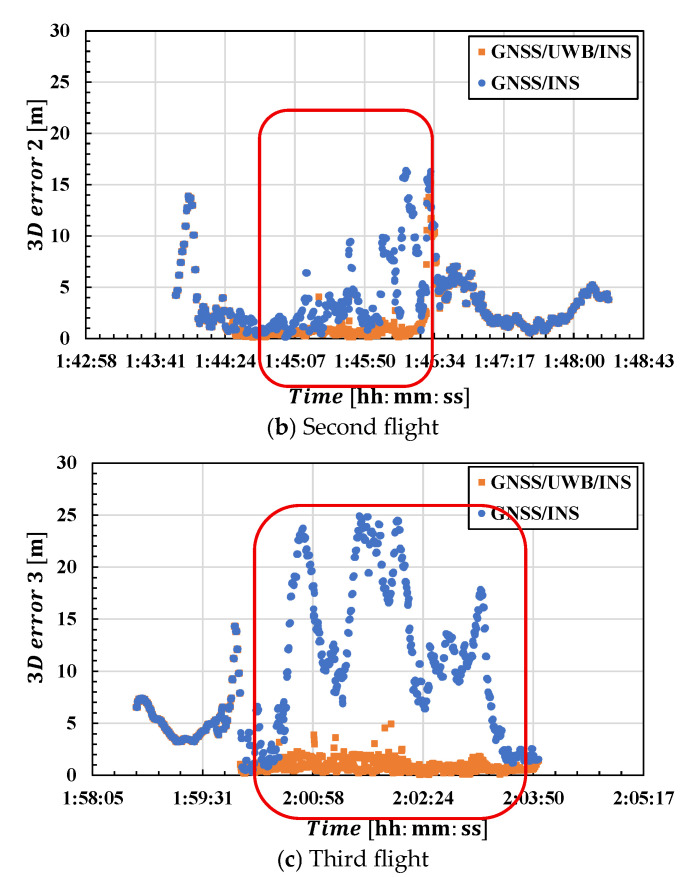
Three-dimensional error for (**a**) first, (**b**) second, and (**c**) third flights. UWB positioning was achieved in the period outlined in red. Blue indicates GNSS/INS; orange indicates GNSS/UWB/INS.

**Figure 17:**
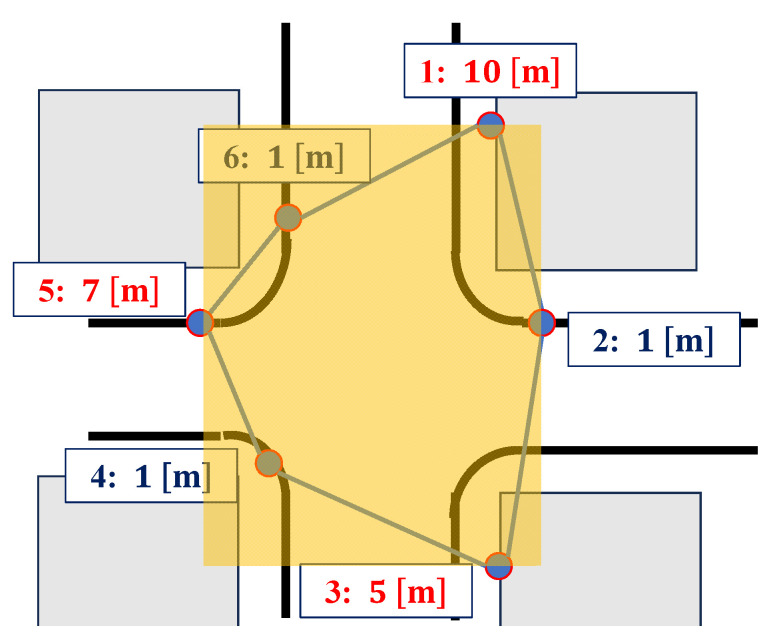
UWB-Anchored Area (the area filled in yellow).

Next, we examine the errors of each integrated navigation system in detail. Figure 16 shows the 3D error for each flight. UWB positioning was performed in the period outlined in red, with the third flight lasting the longest. For the GNSS/UWB/INS plots outside this timeframe, GNSS/INS-integrated positioning or standalone INS was performed. Table 6 shows the RMSE and maximum error comparison between GNSS/INS and GNSS/UWB/INS. Here, the errors of both integration methods were calculated within the UWB-Anchored Area, where $lat_{3}\;<lat<\;lat_{1}$ and $lon_{5}\;<\;lon<lon_{2}$, as shown in Figure 17. The subscripts indicate the anchor numbers. Therefore, the data used for error calculation does not correspond to the data obtained when UWB data was acquired in Figure 10 (data within the red frame). Except for the first flight, most results indicate that UWB fusion is effective for error reduction. Notably, positioning errors during the third flight remained below 1 m throughout the entire flight duration. This is likely due to the longer flight time within the UWB-Anchored Area compared to the first and second flights. In the vertical direction, the effectiveness of the UWB integration was also confirmed. Due to the inherent characteristics of GNSS, the positioning errors in the vertical direction tend to be larger. Consequently, the rate of error reduction achieved through UWB integration was greater than that in the horizontal direction, confirming its effectiveness. Regarding the results of the first flight, as shown in Figure 16a, it is considered that the positioning error did not change significantly compared to the GNSS/INS case because the GNSS accuracy at the time UWB positioning was performed was not particularly poor, and UWB positioning was not performed at the time when positioning accuracy deteriorated.

Table 6 also compares the maximum positioning error. In general, UWB fusion reduced maximum errors compared with GNSS/INS integration. For instance, in the third flight, where GNSS positioning results were unstable, the maximum errors decreased by approximately 80%. However, an exception occurred during the first flight, in which the maximum horizontal error increased by 80%; this point will be discussed later. These results suggest that densely deploying UWB devices in environments with poor GNSS reception, such as underground areas or near high-rise buildings, can provide navigation that is more robust to environmental changes.

**Figure 18:**
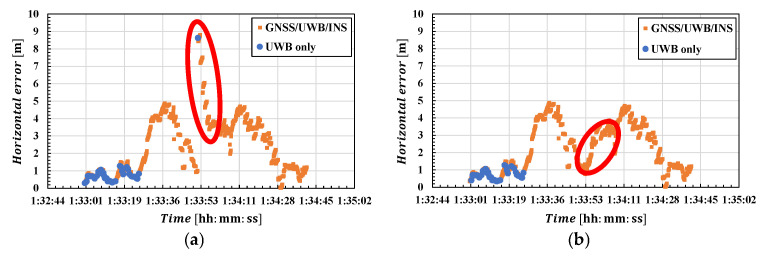
Horizontal positioning error for the first flight: (**a**) before NLOS exclusion; (**b**) after NLOS exclusion.

We now consider the cause of the increased error during the first flight. The left side of Figure 18 shows a portion of the plot of horizontal positioning errors for GNSS/UWB/INS and UWB-only positioning during the first flight. At the time indicated by the red circle in the figure, the UWB-only positioning error temporarily surged sharply. At that time, UWB NLOS waves were observed, leading to significant errors in UWB-only positioning and the integrated positioning solution. In the post-outlier positioning solution, errors remained large for some time due to the influence of NLOS. The right side of Figure 18 shows the positioning error after excluding NLOS data, with areas significantly affected by the exclusion circled in red. It can be confirmed that outliers were eliminated by excluding NLOS data. Table 7 shows a comparison of GNSS/UWB/INS-integrated results before and after NLOS exclusion. In both horizontal and vertical direction, the exclusion of NLOS points resulted in improvements in positioning error and maximum error. While the improvement rate for positioning RMSE was less than 10%, the maximum horizontal error showed a significant improvement, as shown in Figure 18, achieving an improvement rate of approximately 45% in the horizontal direction. During the first flight, significant horizontal errors occurred due to NLOS, so excluding NLOS waves helped reduce these errors. However, excluding NLOS waves also reduces the amount of data, which could potentially compromise positioning stability. Careful consideration based on the positioning conditions is necessary.

Furthermore, Table 8 shows a comparison of UWB positioning errors when using anchor positions obtained directly from GNSS versus those obtained by the inverted UWB described in Section 2. By utilizing anchor positions obtained through the inverted UWB method, we achieved an accuracy of less than 50 cm in both the horizontal and vertical directions. Since the anchor positions are calculated from UWB distance data using reference data as the true values, it might seem natural that the error in the UWB positioning results obtained using anchor positions from the same experiment would be small. However, the positioning results from the first to third trials were all calculated using the anchor positions obtained from the third trial. Even when using anchor positions from different experiments, high-accuracy positioning was achieved, as seen in the results from the first and second trials.

## 6. Conclusions

In this study, we proposed a loosely coupled GNSS/UWB/INS navigation system with anchor position estimation to improve positioning accuracy in multipath-prone urban environments. Outdoor flight experiments demonstrated that the proposed method reduced positioning errors by up to approximately 90% compared with conventional GNSS/INS integration. Specifically, by estimating anchor positions without relying on pre-survey coordinates, we achieved submeter-level positioning accuracy for standalone UWB positioning within the UWB-Anchored Area. These results highlight that anchor position estimation for the inverted UWB is an effective approach to overcome one of the major limitations of UWB systems and greatly enhances flexibility in its deployment. These findings indicate that the proposed method can contribute to safe and reliable navigation solutions for future eVTOL takeoff and landing operations.

Future work will focus on extending this framework to tightly coupled integration to further enhance positioning robustness under challenging urban conditions. This enables the direct use of pseudo-range measurements from GNSS or ranging values from UWB for position estimation, allowing the system to remain effective even when unable to communicate with four or more satellites or anchors simultaneously. In this experiment, we acquired acceleration and attitude angle data using the relatively high-precision ellipse-e INS. However, in practice, it is conceivable that lower-cost, lower-precision hardware could be used. Future plans include verifying the adaptability of the algorithms when low-cost INS is installed on drones, followed by advancing the implementation of a more robust Kalman filter.

The analysis of experimental data in this paper revealed that UWB NLOS signals affect positioning accuracy, and we demonstrated improvements by removing the NLOS signal. However, since NLOS removal was performed during post-flight analysis, a real-time NLOS detection algorithm is required for practical implementation. Since NLOS can occur for each individual anchor, we plan to incorporate this algorithm into the previously mentioned tightly coupled integration method instead of the loosely coupled integration method used in this paper. We will develop an algorithm to detect NLOS based on the quality of the UWB signal, then either remove NLOS signals or apply weighting based on signal quality before performing integration. To ensure real-time capabilities, we plan to apply a Kalman filter type for the fusion filter.

Regarding anchor estimation, we will also examine how the convergence time of estimated positions changes when all anchors are estimated simultaneously. This approach is expected to enable sufficiently accurate estimation of the anchor position even during shorter flights. Regarding the calculation method, we currently use the least squares method with the Levenberg–Marquardt algorithm, but we are considering implementing a Kalman filter. This will allow us to observe the temporal convergence pattern from the covariance values. For the flight paths, we conducted a total of three types of experiments this time. In future research, we plan to explore various paths, including those with significant altitude changes, to establish criteria for paths that are useful for anchor position convergence. Furthermore, while this study assumes the installation of vertiports in multipath environments within urban areas, GNSS becomes virtually unusable when considering challenging NLOS environments, or underground or indoor installations. Therefore, it is considered necessary to explore alternative anchor position estimation methods to replace the proposed technique.

## Figures and Tables

**Table 1:** UERE parameters.

Parameter	$s_{UERE}^{GNSS}\left[m\right]$	$s_{UERE}^{UWB}\left[m\right]$
Value	$\begin{array}{c} 15\;(PDOP>5.0)\\ 10\;(PDOP>3.0)\\ 8\;(PDOP\leq 3.0) \end{array}$	0.1

**Table 2:** Anchor position estimated by the inverted UWB method (third flight).

No.	Latitude [Deg]	Longitude [Deg]	Altitude [m]
1	37.63112978	141.01495082	53.87
2	37.63102664	141.01497757	43.89
3	37.63090325	141.01494878	45.52
4	37.63095534	141.01479009	43.32
5	37.63103971	141.01473920	46.82
6	37.63110109	141.01480428	46.99

**Table 3:** Anchor position estimation error relative to the anchor position calculated from data for the third flight. This shows the average absolute error for each anchor.

No. Flight	Anchor Position Error Relative to the Anchor Position Calculated from the Third Flight Data		
	E [m]	N [m]	U [m]
1	7.72	0.70	4.18
2	8.32	0.82	3.22

**Table 4:** Tag position estimation error (RMSE) and percentage of the number of solutions obtained using AsteRx-i3 D pro+ and mosaic-go compared to PwrPak7 positioning solutions.

Receiver	Tag Pos. Error (RMSE) [m]		Percentage of Number of Solutions Relative to PwrPak7 Data [%]
	Horizontal	Vertical	
AsteRx-i3 D pro+	6.04	6.65	93.4
mosaic-go	3.29	3.66	58.6

**Table 5:** Anchor position estimation error relative to the anchor position calculated from the PwrPak7 data. This shows the average absolute error for each anchor.

Receiver	Anchor Pos. Error Relative to the Anchor Position Calculated from the PwrPak7 Data		
	E [m]	N [m]	U [m]
AsteRx-i3 D pro+	4.47	2.50	6.25
mosaic-go	2.54	1.14	3.67

**Table 6:** Error comparison between GNSS/INS and GNSS/UWB/INS.

No. Flight	Type	Positioning RMSE [m]		Max Error [m]	
		Horizontal	Vertical	Horizontal	Vertical
1	GNSS/INS	2.00	4.17	4.90	13.94
	GNSS/UWB/INS	2.13	3.93	8.81	13.94
	Improvement [%]	−6.28	5.76	−79.8	0.0
2	GNSS/INS	2.42	5.74	6.29	15.54
	GNSS/UWB/INS	1.63	2.90	5.91	13.97
	Improvement [%]	32.6	49.4	6.0	10.1
3	GNSS/INS	5.26	14.62	8.81	23.58
	GNSS/UWB/INS	0.56	0.70	2.03	4.49
	Improvement [%]	89.4	95.2	77.0	81.0

**Table 7:** Comparison of GNSS/UWB/INS before/after excluding UWB NLOS signal waves.

No. Flight	Type	Positioning RMSE [m]		Max Error [m]	
		Horizontal	Vertical	Horizontal	Vertical
1	Before NLOS exclusion	2.13	3.93	8.81	13.94
	After NLOS exclusion	1.94	3.64	4.87	10.47
	Improvement (After NLOS exclusion vs. before NLOS exclusion) [%]	8.9	7.2	44.7	24.9

**Table 8:** Positioning errors due to differences in anchor positioning methods.

No. Flight	Anchor Position Calculation Method	Positioning RMSE	
		Horizontal [m]	Vertical [m]
1	GNSS	1.19	4.84
	Estimated	0.37	0.64
	Improvement [%]	69.2	86.8
2	GNSS	1.49	3.35
	Estimated	0.41	0.56
	Improvement [%]	72.7	83.3
3	GNSS	1.32	4.54
	Estimated	0.46	0.51
	Improvement [%]	65.2	88.7

## Data Availability

The data supporting the findings of this study are not publicly available because they are part of an internal university research project and are subject to institutional restrictions.

## Abbreviations

The following abbreviations are used in this manuscript:

GNSS-RTK	GNSS–Real-Time Kinematic
AHRS	Attitude Heading Reference System
NLOS	Non-Light Of Sight
